# Cancer incidence and mortality in Serbia 1999–2009

**DOI:** 10.1186/1471-2407-13-18

**Published:** 2013-01-15

**Authors:** Jovan Mihajlović, Petros Pechlivanoglou, Marica Miladinov-Mikov, Snežana Živković, Maarten J Postma

**Affiliations:** 1 Department of PharmacoEpidemiology and PharmacoEconomics (PE2), University of Groningen, Antonius Deusinglaan 1, 9713, AV Groningen, The Netherlands; 2 Department of Epidemiology, Oncology Institute of Vojvodina, Put dr Goldmana 4, 21024, Sremska Kamenica, Serbia; 3 Department of Epidemiology, Medical Faculty, University of Novi Sad, Hajduk Veljkova 3, 21000, Novi Sad, Serbia; 4 Department for prevention and control of non-communicable diseases, Center for Disease Prevention and Control, Institute of Public Health of Serbia “Dr Milan Jovanović-Batut”, Dr Subotića 5, 11000, Belgrade, Serbia

**Keywords:** Cancer, Incidence, Mortality, Serbia, Comparison

## Abstract

**Background:**

Despite the increase in cancer incidence in the last years in Serbia, no nation-wide, population-based cancer epidemiology data have been reported. In this study cancer incidence and mortality rates for Serbia are presented using nation-wide data from two population-based cancer registries. These rates are additionally compared to European and global cancer epidemiology estimates. Finally, predictions on Serbian cancer incidence and mortality rates are provided.

**Methods:**

Cancer incidence and mortality was collected from the cancer registries of Central Serbia and Vojvodina from 1999 to 2009. Using age-specific regression models, we estimated time trends and predictions for cancer incidence and mortality for the following five years (2010–2014). The comparison of Serbian with European and global cancer incidence/mortality rates, adjusted to the world population (ASR-W) was performed using Serbian population-based data and estimates from GLOBOCAN 2008.

**Results:**

Increasing trends in both overall cancer incidence and mortality rates were identified for Serbia. In men, lung cancer showed the highest incidence (ASR-W 2009: 70.8/100,000), followed by colorectal (ASR-W 2009: 39.9/100,000), prostate (ASR-W 2009: 29.1/100,000) and bladder cancer (ASR-W 2009: 16.2/100,000). Breast cancer was the most common form of cancer in women (ASR-W 2009: 70.8/100,000) followed by cervical (ASR-W 2009: 25.5/100,000), colorectal (ASR-W 2009: 21.1/100,000) and lung cancer (ASR-W 2009: 19.4/100,000). Prostate and colorectal cancers have been significantly increasing over the last years in men, while this was also observed for breast cancer incidence and lung cancer mortality in women. In 2008 Serbia had the highest mortality rate from breast cancer (ASR-W 2008: 22.7/100,000), among all European countries while incidence and mortality of cervical, lung and colorectal cancer were well above European estimates.

**Conclusion:**

Cancer incidence and mortality in Serbia has been generally increasing over the past years. For a number of cancer sites, incidence and mortality is alarmingly higher than in the majority of European regions. For this increasing trend to be controlled, the management of risk factors that are present among the Serbian population is necessary. Additionally, prevention and early diagnosis are areas where significant improvements could still be made.

## Background

Cancer is the leading cause of mortality in the developed world and the second leading cause in the developing world [1]. However, systematic data collection in cancer epidemiology over the last 30 years has facilitated the assessment and control of the disease on a global level [2-4]. More recently, cancer reporting and monitoring projects, such as the GLOBOCAN 2008, have provided both region- and country-specific estimates of the burden of cancer [5]. Where country-specific data were not readily available, these projects would frequently estimate national incidence and mortality rates using incomplete or indirect evidence. Given the lack of complete data for Serbia, the estimates of incidence have been so far based on local cancer registries and extrapolated to the nation’s population [5].

The first cancer registries in Serbia were formed in 1970, but became obligatory practice after a change of legal acts in 1986 [6]. Since their formation, information was collected separately by the two cancer registries of Serbia: the Cancer Registry of Central Serbia (CRCS) [6] and the Cancer Registry of Vojvodina (CRV) [unpublished data – Miladinov-Mikov]. In the period from 1986 to 1998, quality of data collection from these two registries was rather poor, but, in 1998 they both became members of the International Agency for Research on Cancer and a new methodology has been applied which substantially improved data quality [6]. Although together these two registries monitor the whole Serbian population (excluding Kosovo and Metohija), they have never published cancer estimates on a national level. Consequently, epidemiological studies presented in the literature either relied on one of the registries, were focused on specific cancer sites, referred to estimates from earlier periods or only reported data for smaller time intervals [7-9].

The aim of this work is to present nation-wide Serbian cancer incidence and mortality rates using population-based data and to analyse them with respect to the global and European cancer incidence and mortality rates. Additionally, future predictions until the year 2014 on Serbian cancer incidence and mortality rates are also provided. According to the authors’ knowledge, this is the first time that such nation-wide population-based cancer data are presented for Serbia. This study is expected to enable the evaluation of cancer burden on the society and help decision makers in planning oncological preventive and curative health care.

## Methods

### Data sources

Local incidence and mortality data were extracted from the CRV and the CRCS, for the period between 1999 and 2009 [unpublished data - Miladinov-Mikov, 6]. Information on incidence and mortality were provided by the registries in age-stratified format in five-year age groups. Validity of the collected data was assessed through the percentage of microscopically (histologically) verified cancers (MV%) and the percentage of cancers certified only after death (DCO%) [10]; completeness was assessed through the mortality/incidence ratio (M/I) [11] (Table 1). Malignant tumours were coded according to the Tenth Revision of International Classification of Diseases (codes C00-C96) [12]. Demographical data were collected from the census in 2002 and estimates of the Statistical Office of the Republic of Serbia, for all other years of the study period [13].

**Table 1:** Data quality indicators for cancer registry of central Serbia and cancer registry of Vojvodina (1999–2009)

	**Cancer Registry of Central Serbia**			**Cancer Registry of Vojvodina**		
**Year of report**	**DCO%**	**MV%**	**M/I**	**DCO%**	**MV%**	**M/I**
1999	15.4	68.3	62.7	8.0	52.5	66.5
2000	12.3	69.8	57.2	N/A	N/A	69.4
2001	2.3	N/A	54.4	N/A	N/A	60.1
2002	3.1	92.6	54.7	N/A	N/A	61.8
2003	5.7	91.4	56.3	N/A	N/A	62.9
2004	5.5	88.4	55.4	N/A	N/A	60.0
2005	5.5	87.2	57.5	N/A	N/A	59.1
2006	2.9	86.8	57.4	N/A	N/A	58.5
2007	5.3	84.0	56.0	N/A	N/A	61.0
2008	5.4	87.1	58.1	4.5	84.6	57.0
2009	N/A	N/A	56.4	N/A	N/A	57.5

Legend: DCO% - percentage of cancers certified only after death, MV% – percentage of microscopically verified cancers, M/I mortality/incidence ratio, N/A – data not available.

In order to standardise the incidence and mortality rates to the world population (age-standardised rates on world population - ASR-W), the following procedure was applied for the ten most common cancer sites in each year. Firstly, we aggregated all new cases/deaths from the two registries per age group. Secondly, we divided these with the age-stratified population estimates for every year, thus calculating the age-specific incidence and mortality rates. Finally, direct standardisation with the world population was performed as defined by Segi et al. [14] and incidence and mortality ASR-Ws were obtained for the whole of Serbia.

### Predictions and trends

Next to descriptive information on cancer epidemiology, we aimed to provide predictions of cancer incidence and mortality. Since the quality of registration of new cases has been gradually improving through time, as also shown in Table 1, incidence rates reported in the earlier years (1999–2000) might be less useful for explaining the present and predicting future rates. For this reason, we utilized a prediction method which selectively chooses the most informative sample period, in order to predict incidence/mortality ASR-Ws for the years between 2010 and 2014 [15]. In particular, we used two different regression models: (i) one assuming a linear, age-specific change of incidence/mortality rate over time and (ii) one assuming a linear, age-specific relation between time and the logarithm of incidence/mortality ASR-Ws [15]. These models were used to predict the incidence/mortality rate for a target year, decided to be the latest year of the study period (2009). The former model focuses on the absolute change of incidence or mortality rate while the latter focuses on the proportional change of the rate. Hence, interpretation of the regression parameters should be done cautiously for each model.

In order to identify the most informative sample period for this prediction, we applied both models using information on incidence/mortality from the last four years before 2009 (i.e.2005:2008) and gradually added previous years one by one, up until 2001. This procedure resulted in 2×5 prediction models for age-specific incidence and mortality accordingly. The choice of the appropriate model was then based on the calculated standardized incidence and mortality ratios of the recorded versus the predicted number of total cancer cases or deaths for the target year (2009). The implicit assumption in this method is that the model that makes the best prediction for the most recent available year will also make the best prediction for the future years. After the best fitting model was chosen, predictions for the years 2010–2014 were made. The same model was also used for the estimation of the trend of increase or decrease in the incidence/mortality ASR-Ws through time. All computation within the analysis was conducted using the statistical software R (v.2.13.2) [16].

### Comparison with the GLOBOCAN 2008 data

Contrasting the incidence and mortality of cancer in Serbia with that in other countries and regions in Europe, as well as worldwide, can establish the relative burden of cancer in Serbia. For that reason we compared the incidence and mortality ASR-Ws from the GLOBOCAN 2008 project [5] with the corresponding rates from Serbia in 2008. Site-specific cancer incidence and mortality ASR-Ws were extracted from the Serbian cancer registries and from GLOBOCAN 2008 for the European countries with the highest and lowest incidence/mortality, all European regions (Southern, Central-eastern, Western and Northern), and the world in total. It should be noted that according to the regional division in GLOBOCAN 2008, Serbia belonged to the countries of Southern Europe [17].

## Results

Table 2 presents the number of new cases and the incidence rates for the ten most common cancer sites as well as for all cancer sites combined from 1999 to 2009. In 1999 there were 26,121 incident cancer cases in Serbia (ASR-W: 209.9/100,000, ASR-W in men: 228.9/100,000, ASR-W in women: 197.5/100,000), while this increased to 36,308 newly diagnosed with cancer in 2009 (ASR-W: 277.2/100,000, ASR-W in men: 307.2/100,000, ASR-W in women: 256.4/100,000). For men, lung cancer had the highest incidence rate varying from ASR-W 55.5/100,000 in 1999 to ASR-W 70.8/100,000 in 2009. This was followed by the incidence of colorectal (ASR-W 1999–2009: 27.5/100,000-39.9/100,000), prostate (ASR-W 1999–2009: 12.2/100,000-29.5/100,000) and bladder cancer (ASR-W 1999–2009: 11.7/100,000-16.2/100,000). Among women the most frequently observed cancer in 2009 was breast (ASR-W 1999–2009: 51.5/100,000-70.8/100,000), followed by cervical (ASR-W 1999–2009: 22.7/100,000-25.5/100,000), colorectal (ASR-W 1999–2009: 16.4/100,000-21.1/100,000) and lung cancer (ASR-W 1999–2009: 12.0/100,000-19.4/100,000).

**Table 2:** Cancer incidence and number of new cancer cases in Serbia from 1999 to 2009

		**Number of new cancer cases and incidence ASR-Ws/100,000, male population**										
		**Year of report**										
**Cancer site**		**1999**	**2000**	**2001**	**2002**	**2003**	**2004**	**2005**	**2006**	**2007**	**2008**	**2009**
Lung and bronchus (C34)	Cases	3,281	3,621	4,135	4,131	4,092	4,139	3,992	4,044	3,795	4,193	4,354
	ASR-W	55.5	60.7	69.0	67.6	67.2	67.9	65.4	66.7	61.3	68.0	70.8
Colon and rectum (C18-C20)	Cases	1,690	1,729	1,953	2,130	2,166	2,202	2,240	2,094	2,459	2,698	2,566
	ASR-W	27.5	28.1	31.6	33.5	34.2	34.6	35.1	32.8	38.7	41.5	39.9
Prostate (C61)	Cases	813	973	1,127	1,279	1,333	1,354	1,501	1,662	1,776	1,891	2,193
	ASR-W	12.2	14.4	16.3	17.4	18.9	19.1	20.9	22.9	23.9	25.9	29.5
Bladder (C67)	Cases	733	890	955	1,044	951	1,000	1,009	1,045	1,126	1,044	1,071
	ASR-W	11.7	14.4	14.9	16.0	14.8	15.5	15.8	15.7	16.8	15.6	16.2
Stomach (C16)	Cases	801	837	908	972	857	840	789	747	890	855	729
	ASR-W	13.3	13.6	14.8	15.4	13.6	13.1	12.4	11.9	13.9	13.2	11.1
Larynx (C32)	Cases	604	677	721	709	679	665	618	732	677	661	626
	ASR-W	10.5	11.7	12.4	12.1	11.6	11.2	10.6	12.5	11.4	11.2	10.6
Brain (C71)	Cases	349	378	377	414	465	473	396	423	413	393	396
	ASR-W	7.1	8.0	7.9	8.5	9.3	9.9	7.7	8.6	8.0	7.9	7.9
Mouth and pharynx (C00-C10)	Cases	430	532	494	474	468	457	454	473	435	516	493
	ASR-W	7.5	9.3	8.6	8.1	8.0	7.7	7.7	7.9	7.3	8.6	8.2
Leukemias (C91-C95)	Cases	302	304	332	339	386	335	348	308	405	309	318
	ASR-W	6.5	6.9	7.0	6.9	8.0	6.7	6.8	6.2	7.4	5.8	6.6
Pancreas (C25)	Cases	326	337	432	435	399	458	434	383	457	476	450
	ASR-W	5.4	5.6	7.0	7.0	6.3	7.5	7.0	6.1	7.4	7.3	7.1
**All sites (C00-C97)**	Cases	13,344	15,062	16,128	16,670	16,617	17,049	16,943	17,349	17,801	18,565	19,076
	ASR-W	228.9	255.8	272.8	274.3	276.0	283.2	277.5	284.0	286.3	296.1	307.2
		**Number of new cancer cases and incidence ASR-Ws/100,000, female population**										
		**Year of report**										
**Cancer site**		**1999**	**2000**	**2001**	**2002**	**2003**	**2004**	**2005**	**2006**	**2007**	**2008**	**2009**
Breast (C50)	Cases	3,193	3,692	3,807	3,935	3,866	3,625	3,650	3,791	3,865	4,026	4,518
	ASR-W	51.5	59.3	60.4	63.0	60.2	56.9	57.1	58.9	59.9	62.0	70.8
Cervix uteri (C53)	Cases	1,271	1,341	1,376	1,380	1,287	1,236	1,276	1,392	1,183	1,276	1,423
	ASR-W	22.7	24.1	25.6	25.1	23.2	22.7	23.3	24.9	21.1	23.0	25.5
Colon and rectum (C18-C20)	Cases	1,203	1,241	1,382	1,492	1,549	1,575	1,591	1,595	1,768	1,642	1,614
	ASR-W	16.4	16.8	18.7	19.6	20.3	20.9	20.8	20.9	23.1	21.1	21.1
Lung and bronchus (C34)	Cases	808	1,026	1,114	1,172	1,178	1,288	1,178	1,173	1,152	1,358	1,383
	ASR-W	12.0	15.3	16.4	16.9	16.7	18.3	17.0	16.8	16.1	19.5	19.4
Corpus uteri (C54)	Cases	763	829	767	772	829	827	869	823	844	895	954
	ASR-W	11.7	12.4	11.9	11.7	12.0	12.5	12.5	12.2	12.5	13.1	14.1
Ovary (C56)	Cases	595	641	623	641	606	638	694	602	616	652	626
	ASR-W	9.8	10.5	10.0	10.6	9.8	10.4	11.5	9.8	9.8	10.9	10.3
Stomach (C16)	Cases	404	468	463	502	512	561	493	492	507	482	413
	ASR-W	5.3	6.3	6.2	6.5	6.3	7.1	6.6	6.3	6.5	6.1	5.2
Brain (C71)	Cases	232	288	301	317	315	370	308	310	329	310	330
	ASR-W	4.5	5.9	6.0	6.0	6.1	6.9	5.3	5.8	5.9	5.4	6.2
Leukaemias (C91-C95)	Cases	215	213	259	270	263	252	253	225	294	186	205
	ASR-W	4.1	3.9	4.7	5.1	5.1	4.6	4.6	4.0	4.9	3.2	3.7
Pancreas (C25)	Cases	265	270	289	331	337	349	412	286	347	328	357
	ASR-W	3.5	3.5	3.7	4.3	4.2	4.3	5.0	3.7	4.1	3.9	4.4
**All sites (C00-C97)**	Cases	12,777	14,655	14,943	15,427	15,484	15,786	15,684	15,817	15,923	16,666	17,232
	ASR-W		197.5	225.9	230.6	235.7	233.6	236.9	233.8	235.1	232.3	244.3	256.4

Legend: ASR-W – age-standardised rate on world population. Codes in brackets next to presented cancer sites are in accordance with the Tenth Revision of International Classification of Diseases (codes C00-C96).

Table 3 presents the mortality rates and the number of fatal cases, from 1999 to 2009, for the ten most common cancer sites and for all sites combined. In Serbia 1999, 17,375 people died from cancer (ASR-W: 129.6/100,000, ASR-W in men: 163.2/100,000, ASR-W in women: 102.7/100,000) while the number inclined to 21,069 cancer deaths in 2009 (ASR-W: 143.8/100,000, ASR-W in men: 181.1/100,000, ASR-W in women: 113.8/100,000). The highest mortality rate for men was observed for lung cancer (ASR-W 1999–2009: 48.5/100,000-58.0/100,000) followed by colorectal (ASR-W 1999–2009: 17.8/100,000-21.9/100,000), stomach (ASR-W 1999–2009: 12/100,000-10.8/100,000) and prostate cancer (ASR-W 1999–2009: 9.1/100,000-11.5/100,000). In women, the highest mortality rates were attributed to breast (ASR-W 1999–2009: 22.3/100,000-22.5/100,000), lung (ASR-W 1999–2009: 10.0–16.7/100,000), colorectal (ASR-W 1999–2009: 10.4/100,000-12.0/100,000) and cervical cancer (ASR-W 1999–2009: 7.4/100,000-7.2/100,000).

**Table 3:** Cancer mortality and number of cancer deaths in Serbia from 1999 to 2009

		**Number of cancer deaths and mortality ASR-Ws/100,000, male population**										
		**Year of report**										
**Cancer site**		**1999**	**2000**	**2001**	**2002**	**2003**	**2004**	**2005**	**2006**	**2007**	**2008**	**2009**
Lung and bronchus (C34)	Deaths	2,915	3,054	3,107	3,199	3,212	3,350	3,454	3,647	3,678	3,717	3,676
	ASR-W	48.5	50.4	50.8	51.6	51.9	54.1	55.4	58.2	58.0	59.1	58.0
Colon and rectum (C18-C20)	Deaths	1,124	1,158	1,167	1,244	1,205	1,309	1,383	1,338	1,367	1,438	1,537
	ASR-W	17.8	18.3	18.1	18.8	18.3	19.7	20.8	19.9	19.6	20.8	21.9
Stomach (C16)	Deaths	742	769	767	788	753	787	730	657	713	691	735
	ASR-W	12.0	12.3	12.2	12.0	11.6	11.9	11.1	10.1	10.7	10.4	10.8
Prostate (C61)	Deaths	610	535	605	680	760	759	819	881	948	927	977
	ASR-W	9.1	7.9	8.6	8.9	10.3	10.1	10.5	11.1	11.8	11.5	11.5
Pancreas (C25)	Deaths	403	391	417	435	442	459	502	488	491	534	520
	ASR-W	6.6	6.3	6.6	6.7	6.9	7.2	7.9	7.6	7.4	7.9	7.9
Liver (C22)	Deaths	383	419	389	352	390	374	373	412	468	420	438
	ASR-W	6.1	6.6	6.1	5.4	6.0	5.6	5.7	6.1	6.9	6.3	6.4
Larynx (C32)	Deaths	403	409	384	357	426	366	358	382	366	356	391
	ASR-W	6.7	6.9	6.4	5.9	6.9	5.9	5.6	6.2	5.8	5.6	6.2
Brain (C71)	Deaths	292	295	289	271	327	306	321	338	379	322	331
	ASR-W	5.8	5.8	5.5	5.0	6.0	5.4	6.0	6.4	6.6	5.9	5.7
Bladder (C67)	Deaths	371	344	386	453	417	426	403	495	472	407	453
	ASR-W	5.7	5.3	5.8	6.5	6.0	6.1	5.6	7.0	6.4	5.5	6.0
Leukaemias (C91-C95)	Deaths	238	228	252	230	249	231	279	281	342	293	299
	ASR-W	4.3	4.1	4.5	4.0	4.4	4.0	4.9	4.6	5.7	4.6	4.9
**All sites (C00-C97)**	Deaths	9,870	10,179	10,175	10,635	10,690	10,982	11,169	11,495	11,736	11,775	11,999
	ASR-W	163.2	166.3	164.7	166.6	169.3	172.0	174.2	177.3	179.0	179.6	181.1
		**Number of cancer deaths and mortality ASR-Ws/100,000, female population**										
		**Year of report**										
**Cancer site**		**1999**	**2000**	**2001**	**2002**	**2003**	**2004**	**2005**	**2006**	**2007**	**2008**	**2009**
Breast (C50)	Deaths	1,497		1,511	1,515	1,464	1,608	1,628	1,676	1,643	1,623	1,653	1,674
	ASR-W	22.3	22.6	21.9	20.8	23.0	23.3	23.3	22.2	21.2	22.7	22.5	
Lung and bronchus (C34)	Deaths	712	790	810	857	911	957	980	1,089	1,096	1,107	1,265	
	ASR-W	10.0	11.1	11.2	11.8	12.4	13.0	13.1	14.9	14.5	14.9	16.7	
Colon and rectum (C18-C20)	Deaths	813	845	841	905	937	957	1,070	1,036	990	1,065	1,073	
	ASR-W	10.4	10.4	10.3	10.5	11.3	11.5	12.5	12.1	11.0	12.3	12.0	
Cervix uteri (C53)	Deaths	486	471	490	543	501	497	519	523	523	536	490	
	ASR-W	7.4	7.4	7.7	8.3	7.4	7.6	7.9	8.0	7.7	8.2	7.2	
Stomach (C16)	Deaths	400	436	407	397	424	468	452	393	412	393	417	
	ASR-W	5.0	5.6	5.2	4.8	4.9	5.7	5.5	4.7	4.9	4.6	5.3	
Ovary (C56)	Deaths	335	311	363	331	341	348	374	402	390	388	424	
	ASR-W	4.9	4.7	5.2	4.8	4.9	5.0	5.1	5.8	5.4	5.5	5.9	
Pancreas (C25)	Deaths	306	318	323	349	372	349	448	366	475	435	447	
	ASR-W	3.9	3.9	3.9	4.2	4.5	4.0	5.1	4.3	5.2	4.8	4.8	
Brain (C71)	Deaths	192	206	214	232	227	231	233	281	279	255	292	
	ASR-W	3.2	3.3	3.6	3.9	3.6	3.9	3.6	4.2	4.3	4.0	4.4	
Liver (C22)	Deaths	256	293	275	242	251	278	266	304	312	260	291	
	ASR-W	3.1	3.7	3.3	2.9	3.1	3.1	3.1	3.4	3.6	2.9	3.3	
Leukaemias (C91-C95)	Deaths	195	175	196	186	187	176	175	233	223	215	224	
	ASR-W	3.1	2.6	3.0	2.8	2.7	2.5	2.6	3.5	2.9	2.9	2.9	
**All sites (C00-C97)**	Deaths	7,505	7,694	7,742	7,911	8,169	8,380	8,572	8,722	8,681	8,787	9,070	
	ASR-W	102.7	104.0	103.6	103.7	106.2	108.0	108.8	110.4	108.6	112.7	113.8	

Legend: ASR-W – age-standardised rate on world population. Codes in brackets next to presented cancer sites are in accordance with the Tenth Revision of International Classification of Diseases (codes C00-C96).

### Predictions and trends

**Figure 1:**
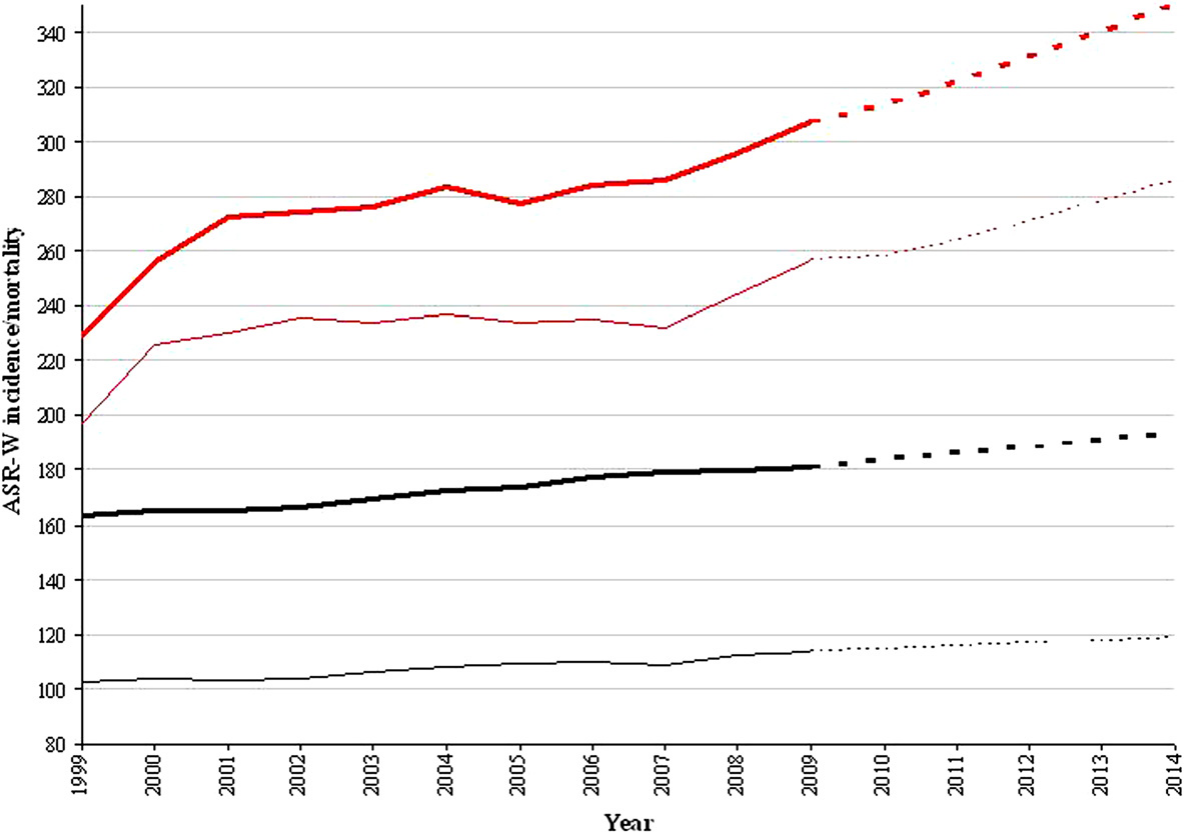
**Overall cancer incidence and mortality in Serbia - trends (1999–2009) and predictions (2010–2014).** Legend: Red bold line – incidence, men. Red regular line – incidence, women. Black bold line – mortality, men. Black regular line, mortality, women. All dotted lines continuous to these are depicting predictions.

Figure 1 presents the overall ASR-W cancer incidence and mortality for men and women from 1999 to 2009 (solid lines). In addition, predictions until 2014 for overall cancer incidence and mortality are presented (dotted lines). An increasing time trend can be observed for cancer incidence and mortality in both genders. In particular, linear regressions of time on the log of mortality and incidence revealed that, in men, incidence and mortality ASR-Ws increased by 2.5% (*P* = 0.004) and 1% (*P* < 0.001) per year, respectively. For women, the incidence ASR-W was estimated to be increasing by 2.22% per year, although this increase was not statistically significant (*P* = 0.067). The cancer mortality rate for women was found to be significantly increasing by 1% (p < 0.001) per year.

**Figure 2:**
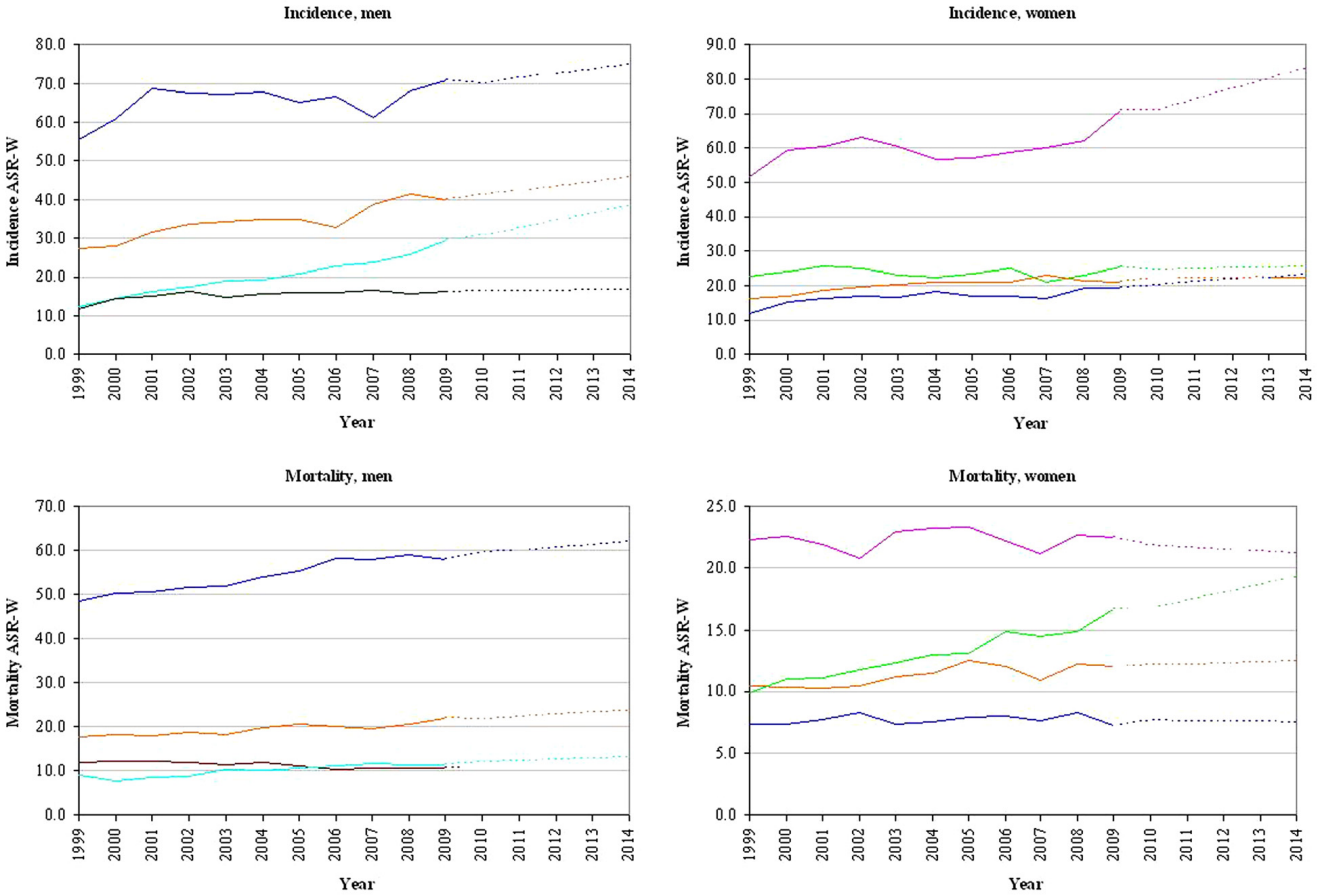
**Trends in incidence and mortality from the four most common cancers in Serbia (1999–2009) and predictions (2010–2014).** Legend: Blue line – lung cancer. Orange line – colorectal cancer. Turquoise line – prostate cancer. Green line – bladder cancer. Dark red line – stomach cancer. Pink line – breast cancer. Light green line – cervical cancer.

Figure 2 presents the time trend for cancer incidence and mortality rates for the four main cancer sites in men and women from 1999 to 2009 as well as incidence and mortality rates’ predictions from 2010 to 2014. Linear regressions of time on incidence revealed that in both genders all cancer sites analysed showed a positive trend over time. In men, the incidence of prostate cancer has been increasing with 1.948/100,000 (*P* < 0.001) per year, faster than any other cancer analysed in the male population. A significant increase was also observed for the incidence ASR-W of colorectal cancer with approximately 1.084/100,000 (*P* = 0.013). In women, breast cancer showed the highest increase in incidence with an additional ASR-W of 3.065/100,000 (*P* = 0.034) every year. In the analysis of mortality ASR-Ws, colorectal cancer in men and lung cancer in women were estimated to have the most significant increase over time: 0.42/100,000 (*P* = 0.036) and 0.626/100,000 (*P* < 0.001) per year, respectively.

### Comparison with the GLOBOCAN 2008 data

**Figure 3:**
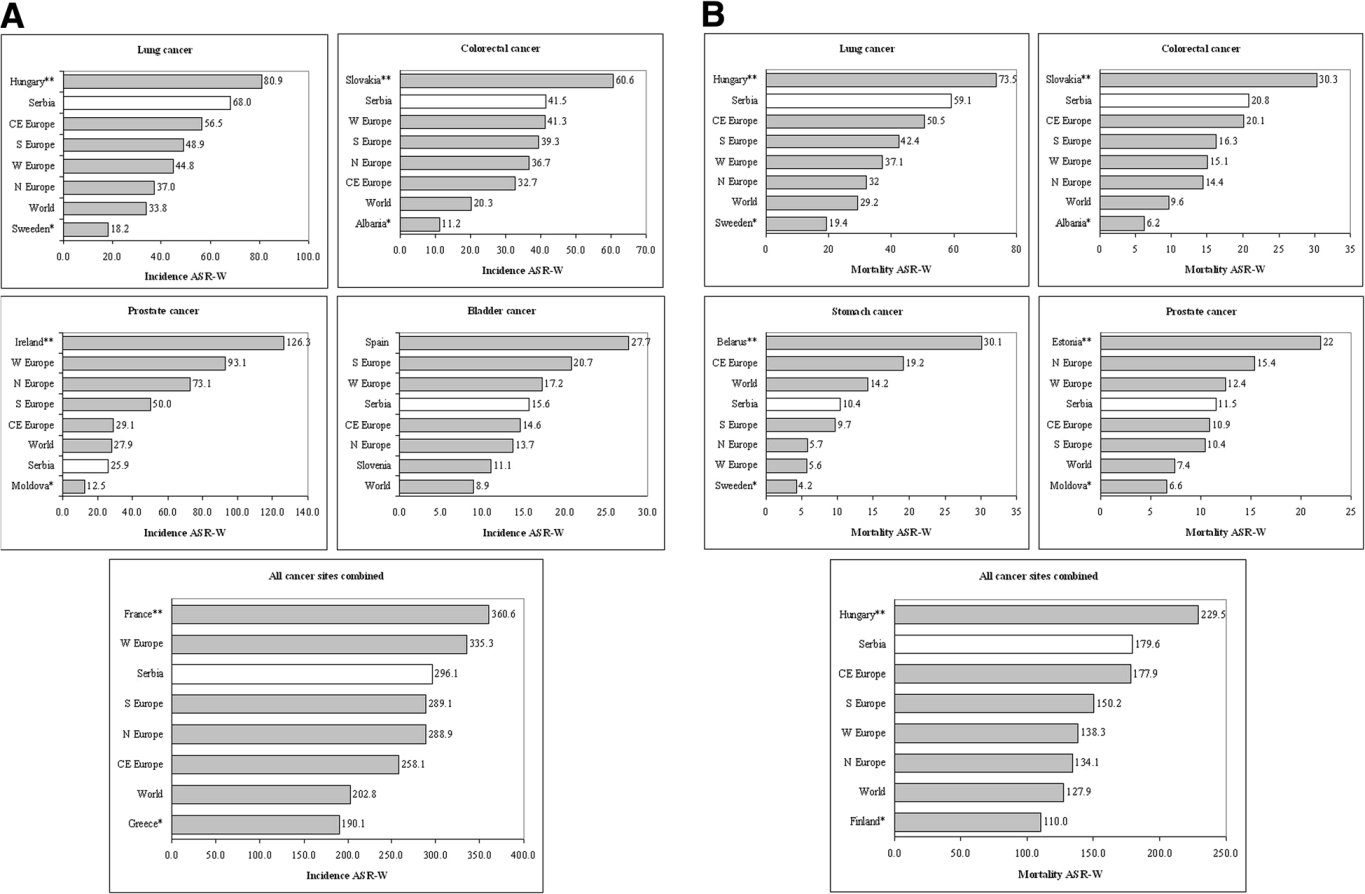
**(A) Comparison of Serbian incidence ASR-Ws with the GLOBOCAN 2008 estimates – men; (B), Comparison of Serbian mortality ASR-Ws with the GLOBOCAN 2008 estimates – men.** N Europe – Northern Europe, S Europe – Southern Europe, W Europe – Western Europe, CE Europe – Central Eastern Europe. * - Eureopan country with the lowest incidence/mortality ASR-W. ** - European country with the highest incidence/mortality ASR-W.

Figure 3A presents the men’s incidence ASR-Ws in Serbia for 2008 against European and worldwide estimates that were extracted from GLOBOCAN 2008. The incidence of lung (ASR-W: 68.0/100,000) and colorectal (ASR-W:41.5/100,000) cancer in Serbia was higher than the average incidence in any European region or in the world. Conversely, prostate cancer in Serbia had a lower incidence rate (ASR-W: 25.9/100,000) than the average in all European regions or the world (ASR-W: 27.9/100,000 – 93.1/100,000). Finally, the overall cancer incidence in the Serbian male population (ASR-W: 296.1/100,000) was closest to the average incidence in Southern Europe (ASR-W: 289.1/100,000). The mortality rates for lung (ASR-W: 59.1/100,000) and colorectal cancer (ASR-W: 20.8/100,000) for men in Serbia were also above the average European and world mortality rates (Figure 3B). Similarly, the overall mortality rate (ASR-W: 179.6/100,000) was higher than world and European averages and comparable to Hungary, the country with the highest cancer mortality in Europe (ASR-W: 229.5/100,000). On the contrary, the mortality rate for prostate cancer (ASR-W: 11.5/100,000) in Serbia was similar to the European average.

**Figure 4:**
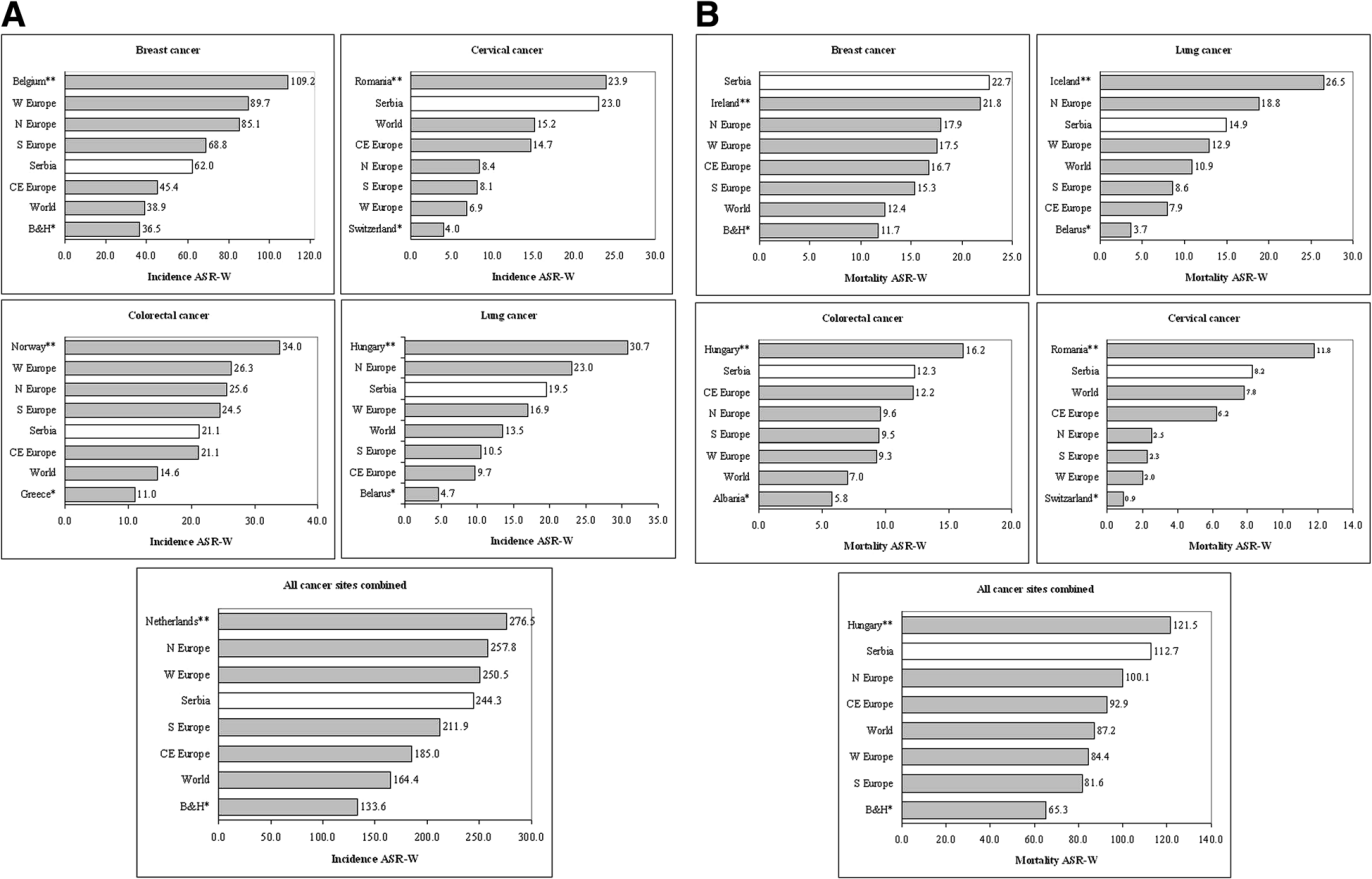
**(A) Comparison of Serbian incidence ASR-Ws with the GLOBOCAN 2008 estimates –women; (B), Comparison of Serbian mortality ASR-Ws with the GLOBOCAN 2008 estimates – women. **N Europe – Northern Europe, S Europe – Southern Europe, W Europe – Western Europe, CE Europe – Central Eastern Europe. B&H – Bosnia and Herzegovina. * - Eureopan country with the lowest incidence/mortality ASR-W. ** - European country with the highest incidence/mortality ASR-W.

The women’s cancer incidence in Serbia, Europe and the world, for 2008, are presented in Figure 4A. The incidence of cervical cancer in Serbia (ASR-W: 23.0/100,000) was observed to be higher than all average European/global estimates and almost equal to the highest European rate found in Romania (ASR-W: 23.9/100,000). The incidence of breast (ASR-W: 62.0/100,000), colorectal (ASR-W: 21.1/100,000) lung (ASR-W: 19.5/100,000) and all cancers combined (ASR-W: 244.3/100,000) in Serbia was close to the average value of the comparators. Finally, comparison of women’s cancer mortality indicated that breast cancer mortality in Serbia (ASR-W: 22.7/100,000) was the highest in Europe in 2008 (Figure 4B). Additionally, mortality of colorectal (ASR-W: 12.3/100,000) cervical cancer (ASR-W: 8.2/100,000) and overall cancer (ASR-W: 112.7/100,000) in Serbian women was above the average regional and global estimates though below the countries with the highest European rates.

## Discussion

This study presents, for the first time, nation-wide data on incidence and mortality from cancer in Serbia over the last decade and a comparison with European and world figures. The main findings of this study are the significant increase of overall cancer incidence and mortality within the observed period in Serbia as well as the alarmingly high mortality rates of Serbia compared to the rest of Europe.

Per-site comparison of incidence and mortality with respective European estimates identified four cancer sites that should attract serious attention. In 2008, Serbia was the country with the highest mortality due to breast cancer in Europe, while incidence of cervical cancer was only slightly lower than the highest European rate, recorded in Romania. In the male population of Serbia, incidence and mortality rates of lung and colorectal cancers, although not the highest in Europe, were well above average estimates. Oppositely, the only rate within the comparison found to be lower in Serbia was prostate cancer incidence.

The comparatively high levels of cancer incidence observed can, to a large extent, be attributed to the prevalence of cancer risk factors. For instance, tobacco and alcohol consumption have a well-established association with increased risk of several cancer types [18,19]. These health hazards are reported as excessively present in contemporary Serbia, where half of all men and one third of all women actively smoke [20], while 40% of the total population consumes alcohol occasionally or on everyday basis [21]. Moreover, risk factors for cancer such as lack of physical activity and obesity [22,23] are also largely present among the Serbian population. In particular, in a representative sample of Serbian adults only a 10% was found to be engaged to a daily physical activity and about 55% were overweight [24]. Although some action in controlling risk factors in the population have been introduced by the Serbian health care system, as for example the creation of a legislation framework for tobacco control [20], more effort towards risk-factor reductions is needed. Finally, the country is still in an economic transition which influences the people’s lifestyle and, as reported, might have negative effect on trends in cancer epidemiology [25].

In addition to this high level of incidence in comparison to the rest of Europe, Serbia is confronted with an even higher relative mortality rate. Since it is known that cancer survival declines with the increase of mortality/incidence ratio [26], cancer survival in Serbia might be poorer than in other European countries/regions. Furthermore, this could also be a sign of delayed cancer diagnosis, which is the main differentiating factor for survival in several cancer types [27]. Lack of screening methods that can facilitate early cancer detection is one of the reasons behind this high relative mortality. Particularly, the opportunistic screening methods available usually do not target the general population, or at least the population at risk. For example, planning of an organized, cytology-based screening for cervical cancer started more than a decade ago [28], yet it still remains on the level of an opportunistic program with high coverage in younger and quite low coverage in elderly women [29]. The implementation of screening programs such as cytology-based screening or mammography could facilitate early detection in cervical and breast cancer respectively and thereby positively impact survival [30,31]. Additionally, improvement in survival after colorectal cancer would be possible through the application of a fecal occult blood test [32]. Hence, prevention and early diagnosis could improve survival and decrease cancer mortality in Serbia.

The trend of incidence of breast cancer in Serbia largely follows the increasing incidence pattern that is currently observed in the rest of the European countries [33]. One reason behind such increase in Serbia is the recent implementation of opportunistic mammography screening [34]. However, breast cancer mortality in Serbia has so far remained constant in contrast to the decreasing trend observed in breast cancer mortality throughout Europe and especially in the northern and western European countries [35]. Since the decrease of breast cancer mortality in Europe has been mainly attributed to advancements in therapy [35], it could be argued that the absence of similar trend in Serbia is caused by delays in the adoption of new treatment alternatives. In men, although prostate cancer incidence in Serbia still remains generally low compared to the rest of Europe, the steep increase of the incidence might be attributed to both lifestyle changes as well as to more widespread use of prostate specific antigen (PSA) testing [36].

Prior epidemiological studies that were conducted for Serbia mainly focused on specific cancer sites. Most of them pinpointed the increasing cancer incidence and mortality [7-9,28,37]. More specifically, high cervical cancer incidence relative to other European countries was noted already at the beginning of the last decade [28,37], the insufficiency of screening utilization was identified as the main cause for this excess in incidence. Increasing trends in breast and lung cancer in Vojvodina were observed in the periods from 1987 until 2001 and from 1996 until 2005, respectively [7,8]. Finally, one study estimated the burden of several cancers in Serbia relative to that in Europe for the year 2000 [9]. This study relied on local data from both cancer registries of Serbia and identified the considerably higher burden of the disease in Serbia.

Our study is also confronted with a number of limitations. A major limitation is the absence of nation-wide cancer survival and prevalence data. Survival and prevalence estimates are typically included in nation-wide cancer epidemiology studies, yet this information is not available for Serbia. Such estimates would require frequent follow up on patient cohorts, something that, due to financial constraints, would be nearly impossible in Serbia nowadays. Moreover, comparisons across Europe were done only with the countries that had the highest and lowest incidence and mortality rates, in order to gain insight on the magnitude of a certain ASR-W in Europe. Additionally, the lack of age-specific cancer incidence and mortality estimates from GLOBOCAN 2008 did not permit a formal statistical comparison between the ASR-Ws of Serbia and the other European regions and countries. In a more detailed analysis, presentation of age-specific cancer evidence from a larger number of European countries could give more precisely the relative position of Serbia with respect to its cancer epidemiology.

## Conclusion

Incidence and mortality of all cancer sites combined in Serbia have shown a steady increase in the first decade of their systematic reporting. Overall mortality rates in both genders appear markedly high relative to European/global estimates. Site-specific comparison revealed alarmingly high rates for four cancer locations: breast and cervical cancer in women and lung and colorectal cancer in men. In light of these findings, we believe that better control of known risk factors should lead to positive change in Serbian cancer epidemiology. Furthermore, prevention and early diagnosis, especially of breast and cervical cancers, seem to be the areas where significant improvements still could be made.

## Competing interests

The authors declare that they have no competing interests.

## Authors’ contributions

JM participated in the acquisition, analysis and interpretation of data, drafted the first version and revised the manuscript. PP carried out statistical analysis, participated in analysis and interpretation of data, drafted the first version and revised the manuscript. MMM participated in the acquisition of data and revised the manuscript. SŽ participated in the acquisition of data and revised the manuscript. MP participated in analysis and interpretation of data, drafted the first version and revised the manuscript. All authors have given final approval of the version to be published.

## Pre-publication history

The pre-publication history for this paper can be accessed here:

http://www.biomedcentral.com/1471-2407/13/18/prepub

## References

[B1] Jemal A Bray F Center MM Ferlay J Ward E Forman D Global cancer statistics CA Cancer J Clin 2011 61 2 69 90 10.3322/caac.20107 21296855

[B2] Parkin DM Laara E Muir CS Estimates of worldwide frequency of sixteen major cancers in 1980 Int J Cancer 1988 41 184 197 10.1002/ijc.2910410205 3338870

[B3] Parkin DM Pisani P Ferlay J Estimates of worldwide incidence of eighteen major cancers in 1985 Int J Cancer 1993 54 594 606 10.1002/ijc.2910540413 8514451

[B4] Parkin DM Pisani P Ferlay J Estimates of the worldwide incidence of twenty-five major cancers in 1990 Int J Cancer 1999 80 827 841 10074914 10.1002/(sici)1097-0215(19990315)80:6<827::aid-ijc6>3.0.co;2-p

[B5] Ferlay J Shin HR Bray F Forman D Mathers C Parkin DM GLOBOCAN 2008, cancer incidence and mortality worldwide: IARC CancerBase No. 10 2010 Lyon: International Agency for Research on Cancer http://globocan.iarc.fr

[B6] Miljuš D Vukičević A Živković S Cancer incidence and mortality in central Serbia (1999–2007) 2010 Belgrade: Cancer Registry of Serbia, Institute of Public Health of Serbia “Dr Milan Jovanović-Batut”

[B7] Stankov S Stankov K Descriptive epidemiology of breast cancer in Vojvodina Breast 2011 20 2 192 195 10.1016/j.breast.2010.12.001 21185723

[B8] Petrović V Miladinov-Mikov M Dugandžija T Topographical analyses of lung cancer incidence and mortality in Vojvodina Arch Oncol 2010 18 3 71 10.2298/AOO1003071P

[B9] Vlajinac H Šipetić-Grujičić S Janković S Marinković J Kocev N Marković-Denić L Bjegović V Burden of cancer in Serbia Croat Med J 2006 47 134 141 16489706 PMC2080367

[B10] Bray F Parkin DM Evaluation of data quality in the cancer registry: Principles and methods. Part I: Comparability, validity and timeliness Eur J Cancer 2009 45 747 755 10.1016/j.ejca.2008.11.032 19117750

[B11] Bray F Parkin DM Evaluation of data quality in the cancer registry: principles and methods. Part II: completeness Eur J Cancer 2009 45 756 764 10.1016/j.ejca.2008.11.033 19128954

[B12] World Health Organization International classification of diseases and related health problems, 10th revision Volume 1 http://www.who.int/classifications/icd/en/

[B13] Statistical Office of the Republic of Serbia http://webrzs.stat.gov.rs/Website

[B14] Segi M Cancer mortality for selected sites in 24 countries (1950–57) 1960 Sendai: Tohoku University School of Public Health

[B15] Dyba T Hakulinen T Comparison of different approaches to incidence prediction based on simple interpolation techniques Stat Med 2000 19 13 1741 1752 10.1002/1097-0258(20000715)19:13<1741::AID-SIM496>3.0.CO;2-O 10861775

[B16] R Development Core Team R: A language and environment for statistical computing. R Foundation for Statistical Computing 2009 Austria: Vienna http://www.R-project.org

[B17] Ferlay J Shin HR Bray F Estimates of worldwide burden of cancer in 2008: GLOBOCAN 2008 Int J Cancer 2010 127 2893 2917 10.1002/ijc.25516 21351269

[B18] Sasco AJ Secretana MB Straif K Tobacco smoking and cancer: a brief review of recent epidemiological evidence Lung Cancer 2004 45 Suppl. 2 S3 S9 15552776 10.1016/j.lungcan.2004.07.998

[B19] IARC Working Group IARC Monographs: On the evaluation of coarcinogenic risks to humans 2010 Volume 96 Lyon: Alcohol consumption

[B20] O’Rourke M Djukic J Welcome to Serbia: feel free to smoke Tob Control 2008 17 428 430 10.1136/tc.2008.027425 18827037

[B21] Knežević T Miljuš D Živković S Plavšić V Janković J Savković S Attributable causes of cancer in Serbia in the year 2005 2008 Belgrade: Institute of Public Health of Serbia “Dr Milan Jovanović-Batut

[B22] IARC working group IARC handbooks of cancer prevention 2002 Volume 6 Lyon: Weight control and physical activity

[B23] Renehan AG Tyson M Egger M Body-mass index and incidence of cancer: a systematic review and meta-analysis of prospective observational studies Lancet 2008 371 569 578 10.1016/S0140-6736(08)60269-X 18280327

[B24] Pavlović M Grujić V Oshaug A Simopoulos AP Nutrition and Physical Activity of the Population in Serbia Nutrition and fitness: obesity, the metabolic syndrome, cardiovascular disease, and cancer 2005 Volume 94 Basel: Karger: World Rev Nutr Diet 51 59 10.1159/000088218 16145250

[B25] Döbrossy L Cancer mortality in central–eastern Europe:facts behind the figures Lancet Oncol 2002 3 6 374 381 8 10.1016/S1470-2045(02)00778-7 12107025

[B26] Vostakolaei FA Karim-Kos HE Janssen-Heijnen MLG Visser O Verbeek ALM Kiemeney LALM The validity of the mortality to incidence ratio as a proxy for site-specific cancer survival Eur J Public Health 2011 21 5 573 577 10.1093/eurpub/ckq120 20813895

[B27] Schrijvers CTM Mackenbach JP Lutz JM Quinn MJ Coleman MP Deprivation, stage at diagnosis and cancer survival Int J Cancer 1995 63 3 324 329 10.1002/ijc.2910630303 7591225

[B28] Stanimirović B National program of early detection and treatment of uterine cervical cancer Arch Oncol 2000 8 2 61 64

[B29] Kesić V Poljak M Rogovskaya S Cervical cancer burden and prevention activities in europe Cancer Epidemiol Biomark Prev 2012 21 1423 1433 10.1158/1055-9965.EPI-12-0181 22956728

[B30] Peto J Gilham C Fletcher O Ematthews F The cervical cancer epidemic that screening has prevented in the UK Lancet 2004 364 9430 249 256 10.1016/S0140-6736(04)16674-9 15262102

[B31] Blanchard K Colbert JA Puri D Mammographic screening: Patterns of use and estimated impact on breast carcinoma survival Cancer 2004 101 3 495 507 10.1002/cncr.20392 15274062

[B32] Kronborg O Fenger C Olsen J Randomised study of screening for colorectal cancer with faecal-occult-blood test Lancet 1996 348 9040 1467 1471 10.1016/S0140-6736(96)03430-7 8942774

[B33] Botha JL Bray F Sankila R Parkin DM Breast cancer incidence and mortality trends in 16 European countries Eur J Cancer 2003 39 12 1718 1729 10.1016/S0959-8049(03)00118-7 12888367

[B34] Ciraj-Bjelac O Štimac FD Aranđić KD Brkić H Good reasons to implement quality assurance in nationwide breast cancer screening programs in Croatia and Serbia: Results from a pilot study Eur J Radiology 2011 78 122 128 10.1016/j.ejrad.2009.10.004 19896314

[B35] Bosetti C Bertuccio P Levi F Chatenoud L Negri E La Vecchia C The decline in breast cancer mortality in Europe: An update (to 2009) Breast 2012 21 77 82 10.1016/j.breast.2011.08.001 21906943

[B36] Janković J Sipetić S The rising incidence and mortality of prostate cancer in Belgrade population Coll Antropol 2011 35 2 499 503 21755724

[B37] Arbyn M The burden of cervical cancer in south-east europe at the beginning of the 21st century Coll Antropol 2007 31 Suppl 2 7 10 17600932

